# Progress in Obstetrics

**Published:** 1902-07-26

**Authors:** 


### PROGRESS IN OBSTETRICS.

**Symphysiotomy.**—Dr. R. C. Buish[1] relates the histories of four patients on whom he had performed symphysiotomy on account of difficult labours. The ages of these patients varied from 20 to 25 years. On one patient (diagonal conjugate 9 cm.), who had previously had three futile confinements, Buish performed a permanent operation to enlarge the pelvis (Frank's method), and subsequently she had an easy labour with large twins. One rickety patient (diagonal conjugate 10 cm.) had the operation performed during the first and third confinements, after forceps had failed; her second confinement was spontaneous. One patient with a diagonal conjugate of 8 cm. had her first and second labours at seven months. Her third child was delivered by symphysiotomy after forceps had failed. A fourth patient with a diagonal conjugate of 8·5 cm. had had three difficult labours. Symphysiotomy was performed here also after forceps had failed. Thus four children were born alive, and the fifth died during delivery as a result of delay. Two patients had femoral thrombosis subsequent to the operation; one suffered from incontinence of urine after the operation, but this was subsequently cured. None of the patients suffered from any impairment of gait, and all were able to do their housework within six weeks of the operation. Ayre's subcutaneous method was adopted in the last four operations, as the after-treatment is simpler than after the open method. Reinprecht[2] reports an interesting case of rupture of the symphysis pubis. The patient was admitted into hospital after her seventh pregnancy, suffering from a laceration due to labour; on examination a longitudinal rent was found in the anterior vaginal wall, and through the rent the free border of the right os pubis could be felt, sundered from the interarticular cartilage. The articulation gaped to the extent of an inch and a half, the symphysis pubis was very painful to the touch, and there was a conspicuous gap between the bones superiorly. The separation appears to have occurred at her first labour, in which forceps were used; the following six pregnancies terminated spontaneously, but after each of these labours the patient could not move her lower extremities for some days, and passive movements caused pain in the groin. A. Martin[3] reports a case in which Cæsarian section was performed after symphysiotomy had failed, although there was 1¼ inches separation of the symphysis. In discussing the case he says that it serves as a lesson that unless the head has entered the pelvis symphysiotomy is useless.

**Hyperemesis Gravidarum.**—Enrico Castelli[4] considers the vomiting of pregnancy to be due to two principal causes: (1) To the influence of pregnancy on the central and peripheral nervous system; (2) to a process of autointoxication. He thinks the toxic cause is the most frequent and the most easily treated. During pregnancy there is an augmentation of the toxic products of the intestine. The liver, which, in association with its other functions, is charged with the neutralisation of those toxins, sometimes becomes unable during pregnancy to meet the increasing exigencies of the organism and a state of autointoxication arises. The vomiting of pregnancy is simply one of the most serious symptoms of such intoxication. Acting on the assumption of the truth of these views, he describes his treatment of these cases as causal and symptomatic. With the causal treatment he seeks to diminish the toxin present in the organism, and to stimulate the action of the liver. This he does by repeated enemata of sodium chloride, sodium sulphate, veratrine and water, and the administration of calomel by the mouth. With the symptomatic treatment he seeks to alleviate the vomiting until the causal treatment becomes effective. Condamin[5] also believes that the vomiting of pregnancy is due to an intoxication of the organism, and the rational treatment is to rid the organism of toxins. He has had great success during the last few years by withholding all food from the stomach for eight or ten days, and by the rectal injection of artificial serum. The rectal injections generally caused very little disturbance, but if any irritation resulted the addition of a few drops of laudanum to the injection was often enough to allay it. If the injections cannot be retained the serum may be given subcutaneously, but by this means large doses cannot be administered at such short intervals. The method is applicable to both slight and severe cases, and the combination of rest for the stomach and the flushing of the tissues has been found to allay not only the vomiting but also the troublesome pyloric and epigastric spasm.

**The Use and Abuse of the Forceps.**—Dr. E. W. Ayres,[6] discussing this subject, believes that the forceps are more often applied too soon than too late. He considers that about one case in every twenty or twenty-five is benefited by the proper use of the forceps. Their application presupposes a knowledge of the presentation and position, a fairly accurate estimate of the relative size of the head of the fœtus to the birth canal, and of the amount of available uterine power. An almost unexceptional indication for the application of the forceps is a slowing and weakening of the uterine contractions accompanied with a rise of the maternal pulse to 150, or slowing of the fœtal pulse to below 120. The traction should not last longer than the uterine contractions, and the blades of the instrument should be relaxed at the end of each traction. The forceps should be applied, if possible, in most cases so that one blade is over the posterior parietal eminence, and the other over the anterior malar bone; they should be almost always removed before the delivery of the head, and the patient's limbs extended at the time the head reaches the vulva, because the flexed thighs increase the tension on the outer portion of the perineum. Dr. Hellier[7] advocates the more frequent use of the axis traction forceps. He says the alleged difficulty of application is very easily overcome, and it is quite a mistake to assert that in any way the prognosis to the child is less favourable than when ordinary forceps are employed.

**Cæsarian Section for Eclampsia.**—Sippel[8] discusses the advisability of performing Cæsarian section for eclampsia. Recognising virulent toxæmia to be the cause of eclampsia, he draws attention to the apparent coincidence between the cessation of pregnancy and the cessation of the eclamptic paroxysms. Reasoning from this, he believes that rapid delivery is indicated in these cases. Where the cervix is undilated and labour has not begun, he considers Cæsarian section indicated. He believes it to be a less irritating traumatism than incision and dilatation of the cervix. He admits that many cases are unsuitable, and quotes statistics of 40 cases with 19 maternal recoveries, and of 41 children of which 23 were saved.

**Pregnancy complicated by Epithelioma of the Cervix.**—Dr. R. Sanderson[9] reports an interesting case of pregnancy of four and a half months complicated by epithelioma of the cervix, in which he performed vagino-abdominal hysterectomy. Finding that the epithelioma was operable, he removed the uterus and the growth by a combined vaginal and abdominal operation, without previous induction of labour. He discusses the ethics and the treatment adopted under the following heads:—(1) That when pregnancy and operable cancer of the cervix co-exist the life of the mother is alone to be considered; (2) that anterior to the fourth month of pregnancy vaginal hysterectomy is the orthodox treatment; (3) that after this period the alternative methods are (*a*) induction of labour followed by vaginal hysterectomy, and (*b*) hysterectomy without induction of labour by a combined vaginal and abdominal operation; (4) that the latter of these alternatives, having regard to the improved statistics of abdominal hysterectomy was in this case to be preferred.

[1] Scottish Med. and Surg. Jour., March 1902. [2] Brit. Med. Jour., March 22, 1902. [3] Brit. Med. Jour., Jan. 25, 1902. [4] Med. Rec., March 22, 1902. [5] Brit. Med. Jour., Feb. 22, 1902. [6] Med. Rec., March 8, 1902. [7] Brit. Med. Jour., Dec. 14, 1901. [8] Amer. Jour. of Med. Sci., Dec. 1901. [9] Brit. Med. Jour., Dec. 14, 1901.

